# Effects of therapeutic horsemanship on caregiver stress scores of children with autism

**DOI:** 10.3389/fpsyt.2025.1574448

**Published:** 2025-07-01

**Authors:** Danielle C. Barron, Madison P. Craft, Emily R. Florek, Brianna N. Stanley, Alexis M. Stoner, Nancy A. Paschall, Sarah Newman, Kimberly I. Tumlin

**Affiliations:** ^1^ Preventive Medicine and Public Health, Edward Via College of Osteopathic Medicine, Spartanburg, SC, United States; ^2^ Healing and Learning Through Equine Relationships (HALTER), Spartanburg, SC, United States; ^3^ Center for Innovation in Population Health, College of Public Health, Lexington, KY, United States; ^4^ Department of Athletic Training and Clinical Nutrition, Sports Medicine Research Institute, College of Health Sciences, University of Kentucky, Lexington, KY, United States

**Keywords:** autism, caregiver, stress, therapeutic horsemanship, equine-assisted services (EAS)

## Abstract

**Introduction:**

Caregivers (primarily parents) of children with autism spectrum disorder (autism) report higher levels of stress, burn out and depression when compared to caregivers of children without autism. Interventions which incorporate animals have been efficacious in improving well-being for children with autism; however, investigating how caregivers are affected when their children are involved in such programs are a nascent field of inquiry. The objective of this pilot study is to characterize emotional strain and stress in caregivers of children with autism when their child attended a therapeutic horsemanship (TH) program.

**Methods:**

Thirteen caregiver-child dyads completed the study. Utilizing a mixed methods approach, caregivers completed the Depression Anxiety Stress Scale (DASS-21) questionnaire prior to and upon completion of their child’s participation in TH for a 16-week semester. Semi-structured interviews were performed once during the semester and transcripts were analyzed using thematic analysis.

**Results:**

We observed caregivers experienced a statistically significant decrease (p=0.03) in their stress levels over a single semester of TH participation demonstrated by a reduction in DASS-21 stress subcategory (pre intervention mean 12.77 (SD = 9.95), post-intervention mean 8.62 (SD = 10.98). A total of five common themes were identified from the caregivers’ interview responses. Of these, four were associated with increased caregiver stress and strain: 1) navigating the care and management of their child’s diagnosis; 2) the lack of resources for their child with autism (CWA); 3) managing finances; and 4) caring for more than one child in their household. In contrast, the fifth theme captured a reduction in caregiver stress and strain secondary to their indirect involvement in TH.

**Discussion:**

This pilot study successfully captured the indirect effects of a TH program in caregivers of children with autism. Integral to understanding caregiver stress, this study further characterizes how caregiver emotional stress and strain can be impacted as their child builds life skills in TH.

## Introduction

The Center for Disease Control and Prevention’s Autism and Developmental Disabilities Monitoring (ADDM) Network identified the prevalence of autism to be approximately 1 in 36 children and nearly four times more common in boys for the 2020 surveillance year. This data represents a marked increase from the 2018 surveillance year that reported a prevalence of approximately 1 in every 44 children (1). Children with autism (CWA) have differing social communication and interactions than the neuro-majority population. The World Health Organization recommends a Nurturing Care Framework, which prioritizes positive relationships with caregivers, and supports the ability for CWA to thrive (2, 3). Understanding the impacts on these relationships between a CWA, the caregiver, and therapist is key to ensuring the needs and goals of CWA and their caregivers are met (3). Children with autism often breach social norms in face-to-face interactions and may display antisocial behaviors in the form of withdrawal or aggression (4, 5). These behaviors present unique challenges to caregivers of CWA, which has been demonstrated by increased levels of burnout, stress, and depression as compared to caregivers of children without autism (6, 7). Financial hardship, disruption of routines, career interference, fatigue, and social isolation have also been reported as contributing to increased levels of caregiver and familial strain (8–11). Interventions for CWA often depend on the primary caregivers’ ability to implement strategies outside of the therapeutic setting, which can introduce additional compounding and cyclical negative impacts (11). Many studies have examined different approaches to alleviate caregiver stress and improve psychological well-being independently of their child’s autism (12, 13).

The Professional Association of Therapeutic Horsemanship International (PATH Intl.) is the recognized leader responsible for certifying therapeutic riding instructors. The field of equine-assisted service (EAS) is described as a number of service areas in which professionals incorporate horses to benefit people (14). Broadly, EAS provides opportunities for educational, motivational, and recreational benefits with an overall aim of enhancing the individual’s quality of life (15). Various therapeutic approaches within the realm of EAS have shown positive psychosocial outcomes in CWA (16). TH focuses on building life skills by teaching CWA how to interact with a horse while mounted, meaning that the CWA completes tasks while on the back of horse. While not exclusively completed in a mounted format, TH incorporates a variety of activities that are not therapy (12).

While direct benefits of TH are well documented for CWA, the indirect effects on the psychosocial and physical health in caregivers is nascent field of inquiry (16). Thus, this study aimed to characterize emotional strain and stress in caregivers when their CWA participated in TH.

## Materials and methods

This pilot study utilized a mixed-method longitudinal study design. The Edward Via College of Osteopathic Medicine’s Institutional Review Board reference number 2023-171, package number of 2126547–2 approved the study as exempt. Participants were not charged a fee for participation in the study nor was compensation provided. No external funding was used to complete the study.

### Study population

Eligible participants (primary caregivers of CWA) were initially identified via review of enrollment records from Healing and Learning Through Equine Relationships (HALTER). Children were selected by 1) identifying current enrollment in the therapeutic horsemanship program at HALTER; 2) sorting enrollments as having a medical diagnosis of autism as reported by caregivers; and 3) having current contact information for the caregivers. For CWA, a primary caregiver was defined as having primary responsibility to bring the CWA to weekly sessions. Once identified, these caregivers were recruited using ethical review approved electronic flyers. Caregivers who chose to participate in the study were required to submit completed informed consent by January of 2024. Caregivers who completed and returned the informed consent prior to this date were considered active participants in the study. Non-English-speaking caregivers were excluded from this study, given the inability of the research team to support individuals with English as a second language during recruitment, data collection, and general study communications.

### Study procedures

#### Therapeutic Horsemanship

Caregivers’ children attended one semester of TH. A semester was defined as sixteen weeks, with one-hour structured lessons with horses occurring weekly (i.e., a session). Sessions were conducted at one location in Spartanburg, South Carolina, USA by Certified Therapeutic Horsemanship Instructors (PATH, Int’l). The structured therapeutic horsemanship sessions consisted of pre-determined activities based on lesson plans created by HALTER’s certified instructors. Activities included various games and experiences aimed to develop horsemanship skills, including social, emotional, and communication skills and to provide the healthful benefits of interacting with horses. Both mounted and unmounted activities were utilized during sessions. Examples of these skills are counting strides between two marked spots that the horse and rider combination travel between. Other skills such as steering in circles or from point A to B are examples of mounted activity. Unmounted activity includes brushing the horse, leading the horse, and generally interacting with HALTER instructors to complete horsemanship skills.

#### Data collection

##### Quantitative data

The Depression, Anxiety, and Stress Scale 21(DASS-21) questionnaire was utilized to evaluate caregiver depression, anxiety and stress (**Appendix 1**). As a self-report, the DASS-21 provides separate scores for each subscale, with higher scores reflecting greater symptom severity (17–21). The measure has demonstrated good internal consistency in caregivers of CWA (22). Eapen et al. reported Cronbach’s alpha coefficients supporting its reliability in this specific caregiver population (DASS-21 – Depression: α = 0.86, Anxiety: α = 0.75, Stress: α = 0.81) (22). Moreover, prior research has shown that the DASS-21 possesses moderate sensitivity in detecting elevated psychological distress among caregivers of CWA (6, 23). It has also proven effective in capturing reduction in subscale scores following caregiver and child targeted interventions (21).

Prior to the start of the Spring semester of therapeutic horsemanship services, caregivers completed the DASS-21 questionnaire as a baseline metric. Conducted at the end of Winter break, this assessment was given after the CWA had not actively participated in TH services for approximately four weeks. Upon completion of the child’s sixteen-week course of TH horsemanship, caregivers completed a post- DASS-21 questionnaire.

##### Qualitative data

Semi-structured interviews were conducted through a web-based platform. These interviews were completed once during the child’s sixteen-week course of TH. Interviews were scheduled at the caregivers’ convenience. Each interview consisted of the same ten open-ended questions (**Appendix 2**), developed to assess perceptions of psychosocial improvements and other factors related to stress. The questionnaire was reviewed by an advisory team including research, clinical, and analytical professionals. Questions were evaluated for language which may create bias. Follow-up questions were asked on a case-by-case basis when additional clarification was needed regarding a caregiver’s response to one of the original ten questions. These clarification questions were unstructured. Upon completion of each interview, the audio portion of the recordings was transcribed with children’s names replaced with pseudonyms to maintain confidentiality following the approved ethics protocol.

#### Data analysis

##### Quantitative data

Analyses began by summarizing sample mean and standard deviation for pre DASS-21 scores. Change in DASS-21 scores were calculated as the difference between post measurement to premeasurement. Though 19 families began the study, only 13 families provided both pre and post measurements. Due to the small sample size, the median change score and interquartile range for the change score were calculated. To test for statistically significant change, we the Wilcoxon Signed Rank test using a two tailed Type I error rate of 0.05 was used.

##### Qualitative data

Thematic analysis was conducted using transcripts from caregiver interviews following a systematic and collaborative process. Four members of the research team independently read all interview transcripts in the entirety to become familiar with their content. Each of the four researchers generated initial codes by identifying portions of the data that reflected 1) emotional strain in caregivers of CWA, or 2) related to the child’s participation in TH. The team subsequently met to discuss and compare codes, and collaboratively structured codes into broader categories. Through an iterative process, the team refined these broader categories into overarching themes which collectively reflected data patterns. To ensure consistency, the team revisited the transcripts and initial codes collectively to confirm that each theme was clearly defined and thoroughly supported by the dataset.

## Results

Though 19 caregivers were recruited from the HALTER organization to participate in this pilot study, only thirteen caregiver-child dyads completed the study. Attrition was the result of the following: 1) three caregivers were unable to complete the initial DASS-21 questionnaire before the semester began; 2) one caregiver withdrew from the study which was secondary to withdrawing from TH at HALTER; and 3) two caregivers were not able to complete the semi-structured interview session. Demographics of primary caregivers identified as mothers (n=9), fathers (n=3), and a grandfather (n=1).

### Quantitative data

Using a two tailed Type I error rate of 0.05, caregivers experienced a statistically significant decrease (p=0.03) in their stress levels over a single semester while CWA participated in TH (pre intervention mean 12.77, SD = 9.95; post-intervention mean 8.62, SD = 10.98; see **Table 1**). Neither depression nor anxiety assessments revealed a statistically significant decrease from pre- to post-intervention values (depression (pre-intervention mean 4.31 (SD = 6.92), post-intervention mean 2.31 (SD = 4.49) and anxiety (pre-intervention mean 5.54 (SD = 5.84), post-intervention mean 4.00 (SD = 4.47) see **Table 1**).

**Table 1:** DASS-21 scores.

Measurement	Pre-Mean ± Standard Deviation	Post Mean ± Standard Deviation	Change Mean ± Standard Deviation	Change Median ± Interquartile Range	p-value
Depression	4.31 ± 6.92	2.31 ± 4.49	-2.0 ± 4.0	0.0 ± 6.0	0.12
Anxiety	5.54 ± 5.84	4.00 ± 4.47	-1.54 ± 3.28	0.0 ± 2.0	0.19
Stress	12.77 ± 9.95	8.62 ± 10.97	-4.15 ± 3.69	-4.0 ± 6.0	0.03

DASS-21 Score comparison from parents prior to and following child involvement in equine-assisted services for 16 weeks.

### Qualitative data

Qualitative data analysis revealed four common themes identified by caregivers as specific sources of increased stress associated with caring for CWA. These themes included navigating the care and management of their child’s diagnosis, lack of resources for their child, managing finances, and caring for more than one child in their household. These recurring themes were considered particularly significant in characterizing caregiver stress and emotional burden, as they were most frequently referenced and supported by participant interview data. However, data analysis also revealed that caregiver experiences were not solely defined by stressors; participation in TH emerged as a fifth recurring theme associated with reduced stress and enhanced emotional well-being when caring for a CWA.

### Navigating the care and management of their child’s diagnosis

Caregiver stress related to the navigation and management of their child’s autism diagnosis was reported by 100% of study participants. Each caregiver indicated an increase in their perceived stress level when called upon to make decisions regarding their child’s treatment and further care.

“ *… I worry because I don’t know if I am doing enough [interventions] or if I am doing too little. I worry that there is something else Matthew needs, and I just haven’t found it yet.*”

Most caregivers reported higher levels of stress and emotional strain associated with their child’s future and the uncertainty that it holds. Specifically, caregivers identified that preparing for a future when they were no longer able to serve as the primary caregiver for their child was a significant source of stress.

“*My main stressor is just trying to make sure that Mark is in a place where he will be able to function in the world without me.*”

“*… I am constantly having to make sure Jane is getting acclimated to typical society and can handle those daily interactions, so they are prepared for when I am not there.*”

Additionally, caregivers identified that navigating their child’s growing independence while maintaining an appropriate level of supervision presented both challenges and stress. Similarly, caregivers found it difficult to balance their child’s preferences while still ensuring that their child was receiving appropriate therapeutic interventions.

“*I struggle with John getting older. I know he will need to be more independent, but I don’t know what that looks like, so I struggle with navigating his independence while keeping him safe.*”

“*I try to make sure I am being attentive and listening to Sally’s voice, while also making sure they get the therapy they need.*”

Caregiver stress related to the navigation and management of their child’s diagnosis was the only theme identified and supported by each caregiver in the pilot study emphasizing its importance in contributing to increased stress and emotional strain in caregivers.

### Lack of resources or support for their CWA

Caregiver stress related to a lack of resources or support for their CWA was another theme identified as significantly contributing to increased perceived stress in the study population.

Ninety-two percent of caregivers reported that their child’s medical needs could not be met within their local community. As a result, caregivers reported an average of one hour of travel time to access the medical care their child requires. Caregivers not only described the stress associated with this travel, but also highlighted the shortage of specialized care for CWA in their area. Compounding this issue, many caregivers expressed encountering extensive waitlists when trying to schedule medical care and therapies catering to CWA.

“*It’s very hard to get into occupational therapy and the early interventional program. We do have some resources, but their size doesn’t fully accommodate the children in our community. The waitlists are very long.*”

“*There is a long waitlist for everything here; just trying to get an appointment with the developmental pediatrician is hard. It’s difficult because a lot of the appointments and therapies Casey needs are time sensitive.*”

Furthermore, caregivers reported feeling unsupported by their child’s local education system. Most frequently reported was a lack of communication between the child’s school and medical team. Many caregivers felt that their child’s school system did not understand nor attempt to accommodate the recommendations provided by their child’s medical team.

“*I struggle with getting the correct classes and support for Caleb at school. I feel like I do not have any support or resources there.*”

“*It’s very hard getting [the school system] to understand recommendations from our medical team and even harder getting them to implement them. Support and communication from the school is really lacking.*”

Additionally, caregivers expressed a lack of recreational activities serving CWA within the community.

“*I do not think there is an adequate number of resources for our children with autism in the community … I believe local community leaders need to try and expand that network. Spartanburg is not up to where it should be to support children with autism and other special needs.*”

“*There are not nearly enough resources for children with autism in our area. In the summer, there are all sorts of programs for children without autism, but finding something for Jamie is very difficult.*”

As supported by caregiver data, many experiences characterized as relating to a lack of resources or support for children with autism contribute to increased levels of caregiver stress.

### Managing finances

While not unique to caregivers of CWA, increased stress related to managing finances was reported by 46% of caregivers in the study. Data classified within this theme included financial concerns related to caregiver income and financial concerns related to medical expenses including medical insurance coverage.

“*A significant stressor in my life is dealing with doctors and insurance companies and trying to get the therapy Brooke needs covered by insurance; it’s the only way we can afford the care she needs.*”

*“I think my stressors are regular mom things, like trying to ensure the kids are healthy and safe and our family finances.”*

“*There are times when I would like to enroll James in additional therapies or other activities but then the cost prohibits me from enrolling him.*”

Financial concerns reportedly contributed to caregiver stress, but also had significant impact on caregivers’ ability to provide desired therapies to their children. Caregivers indicated that medical insurance coverage determined which therapies they were able to afford. Caregivers specifically reported heightened stress levels when they were unable to afford a therapeutic activities out-of-pocket, when they thought would be extremely beneficial or helpful to their child.

### Caring for multiple children within the same household

Interview data supported that 38% of caregivers experienced higher levels of perceived stress when responsible for multiple children. Of the caregivers who participated in the study, none reported being a caregiver to more than one child diagnosed with autism. Data within this theme included caregiver reports detailing the challenges of being the primary caregiver for a CWA while also caring for one or more children without the diagnosis. Primarily, caregivers reported heightened stress related to balancing the needs of a CWA while also attending to the needs of a child without autism. Many caregivers expressed feelings of worry and guilt about spending more one-on-one time with their CWA due to the extensive interventions required, often at the expense of time with their other children.

“*I have another child without autism, and they require totally different things than my child with autism so, balancing between both children can be very stressful.*”

“*One of my stressors is being able to focus on my child with special needs while also raising their younger siblings. My youngest children are both in a typical classroom setting as opposed to Jane, who is in a resource class, so keeping up with everybody’s daily schedule and making sure everyone gets the attention they need is very difficult.*”

### Therapeutic Horsemanship participation

All caregivers reported that their child’s participation in TH had a significant impact in reducing their own levels of stress and emotional strain. The data supported a broad range of indirect positive effects associated with their child’s involvement in TH.

“*I am not a member of any specific support group for parents of children with autism, but we do participate in HALTER, so I feel like that is my support group in a way. Though it’s not technically a formal support group, it’s a place where I can talk to other parents that are experiencing the same things as me.*”

“*I have multiple children, but only one with special needs. Most of the time my other children attend therapy sessions for Amy because our schedule doesn’t allow me to take them anywhere else during that time. During the other therapy sessions, I need to bring activities to keep my other children occupied; however, HALTER is really family centered, and I don’t have to worry about my other children during those sessions. During Amy’s class time, there are always other siblings for my children to play with or fun activities for them to do during their sister’s lesson. It’s one small thing I don’t have to worry about, but it makes a big difference.*”

“*HALTER was very helpful while we were trying to navigate my daughter’s diagnosis while moving. When we first moved to the area, it was one of the first activities I could find without a waitlist for Kate. Other therapies had very long waitlist. As we went through the process of transferring all of Kate’s medical care to South Carolina, the other parents at HALTER were able to help with recommendations for doctors and types of therapies. It was helpful to hear the opinions from people who had been in the area and were going through similar things.*”

“*I am a part of a couple of Facebook groups for parents of children with autism, but not all the advice in those groups is very helpful because people from all over the world post. While their advice is helpful for them, it may not be helpful to me and where I live. For example, I was having a really hard time getting all the paperwork for Tim’s IEP and 504 plan with the school. I felt like the school wasn’t listening and I would never be able to get Tim the accommodations he needed. I had posted on one of the Facebook groups and received some advice, but nothing that was helpful to my situation. However, when I talked about my concerns with another parent at Tim’s HALTER lesson, she was able to help me. She had just been through the same process with her child in the same school system and was able help me get the accommodations I needed for Tim. I would have never met that other mom and had the help I needed without HALTER.*”

“*I think HALTER benefits our whole family. My wife volunteers a lot and the people that run the organization are very attentive to each of the families in the program. They are always checking in on us.*”

“*HALTER is very important to us as a family. They are one of the few organizations in our community that is constantly trying to raise awareness and bring our community’s attention to children with challenges. They are never stop advocating for our kids. You know, I often talk to other parents, and we talk about the need to constantly advocate for our own kids with their doctors and therapies and how overwhelming it can feel, but It’s nice to know places like HALTER exist that help us advocate for our kids on a bigger scale.*”

The experience of reduced emotional strain and stress as an indirect result of their child’s participation in TH provided by HALTER proved to be a consistent theme across all caregiver interviews. These indirect benefits frequently included emotional support that extended beyond the individual caregiver to the entire family unit and sharing of resources. Additionally, caregivers expressed a heightened sense of reassurance and support, knowing that HALTER actively advocates for children and families like their own.

## Discussion

This study was successful in capturing the indirect effects of a TH on caregivers of CWA. The importance of this population in care delivery cannot be underestimated. Reductions in measured stress were an indirect impact factor when CWA were participating in TH horsemanship. Identification of potential areas for support that can be provided by TH was a means to help support neurodiversity affirming practices, such as embracing the value that CWA bring to a family system. As the prevalence of autism continues to rise, it is imperative to identify interventions that reduce caregiver stress and create alternative avenues to further support caregivers as they navigate the unique challenges of caring for a CWA (1). This study provides a solid foundation that TH has reach beyond the traditional participant and impacts the family unit through reduction of caregiver stress.

Previous research has theorized that statistically significant improvements in a child’s irritability, hyperactivity, social cognition, and social communication gained by participation in TH programs likely enhance the caregiver-child relationship, contributing to decreased caregiver stress (24). While a child’s progress in the aforementioned areas likely plays a critical role in alleviating caregiver stress, it is equally important to acknowledge that having a structured activity provides the caregivers time to socialize, connecting to others with similar family dynamics, pursue their own physical activity, or have uninterrupted self-care time.

Key constructs which contribute to caregiver stress found in this study included navigating the care and management of their child’s diagnosis, lack of resources specifically tailored for CWA to engage in, managing finances, and caring for more than one child. In more rural communities, the presence of TH provides a nurturing and engaging environment in which CWA can thrive.

Qualitative data from this pilot study suggest that a child’s participation in TH contributes to lower perceived levels of caregiver stress, particularly in relation to managing and navigating their child’s diagnosis. Many caregivers described informal conversations with other caregivers during their child’s lessons functioning like an unstructured support group. These interactions provided opportunities to share resources, strategies, and personal experiences, creating a sense of connection and solidarity among families facing similar challenges. This informal support network, formed as an indirect outcome of participating in TH services, likely contributed significantly to the observed reduction in caregiver stress scores.

Most participants in the study identified a lack of resources or support for their CWA as a significant contributor to increased stress levels. Caregivers specifically identified a shortage of specialized care providers, long waitlist for therapy services, and challenges in coordinating multidisciplinary care. However, caregivers also reported that their child’s participation in TH helped alleviate some of the stress associated with limited community resources. Through their involvement with TH at HALTER, caregivers became more aware of the organization’s advocacy efforts at the local, state, and national levels. Many caregivers expressed a sense of reassurance and support, knowing that others were actively working to advocate for children like theirs. The sense of reassurance and support fostered by HALTER’s advocacy efforts likely had an indirect impact on reducing the emotional strain caregivers experienced due to the lack of resources for their CWA.

The investigators acknowledge that elevated stress levels related to financial management and caring for multiple children are not unique to caregivers of CWA. While these stressors are common among caregivers more broadly, the data from this study suggest that such stress is intensified when caring for a CWA. Notably, findings from this pilot study indicate that participation in TH indirectly helps to alleviate caregiver stress in both areas. HALTER’s scholarship program, funded through donations, helps offset the cost of participation, likely reducing financial strain among caregivers. Additionally, caregivers reported feeling less overwhelmed by the demands of caring for multiple children, as siblings were welcomed at TH and provided with opportunities for engagement, entertainment, and social support during their siblings’ classes.

### Limitations

Although the present study demonstrated improvement in caregiver stress scores associated with their child’s participation in TH, these findings should be interpreted within the context of several limitations. As a pilot study, the sample size was small, limiting the generalizability of the results. The intervention period was confined to a single 16-wk time frame, and no long-term follow-up data were collected to assess the sustainability of the observed outcomes. Additionally, caregiver stress levels were self-reported, introducing the potential for social desirability bias. The pretest-posttest design also presents inherent limitations, as changes in caregiver stress may have been influenced by external factors unrelated to TH participation. Despite these limitations, the study provides valuable insights into a population that is often underrepresented in autism research, provides an avenue for neurodiversity-affirming care, and contributes to the emerging evidence supporting therapeutic horsemanship services to support caregivers.

### Future research

Future research should include a larger and more diverse sample of caregivers utilizing TH across various geographic regions to enhance the generalizability of the findings. Specific elements in the environment of TH requires more detailed investigation, for example, organizational culture, interpersonal interactions, structured support, and integration into horse-focused activities to evaluate how these factors may add to reduction of caregiver stress. Consideration should also be given to external factors that may influence caregiver stress levels by collecting information on what other activities CWA participate in, and what personal-care strategies the caregiver uses to test the independence of TH. To further understand caregiver stress and improve internal validity, future studies should incorporate additional methods of stress measurement beyond self-report, including biometric outcomes which help mitigate social desirability bias.

## Conclusion

This study characterized four common themes underlying caregiver stress responses: 1) navigating the care and management of their child’s diagnosis, 2) limited access to resources and support, 3) financial strain, and 4) challenges of caring for multiple children within the same household. Importantly, this study highlighted a fifth common theme among caregivers who reported lower perceived stress because of the indirect benefits associated with their child’s involvement in TH. These findings emphasize the need for practices to evolve in support of caregivers of CWA by expanding care and activities which are naturally neurodiversity-affirming because of the ability of horses to provide non-judgmental, skills-building opportunities for CWA.

## Data Availability

The raw data supporting the conclusions of this article will be made available by the authors, without undue reservation.

## Supplementary material

Appendix 1 DASS-21 Questionnaire: DASS-21 questionnaire used to gather quantitative data pre- and post-intervention. DASS-21 is an Open Access Document

Appendix 2 Semi-Structured Questions: Questions used gather qualitative data during participants semi-structured interviews.
