# Supplementary material for: Effects of therapeutic horsemanship on caregiver stress scores of children with autism
Source: Front Psychiatry. 2025 Jul 1;16:1574448. doi: 10.3389/fpsyt.2025.1574448 (PMC12261109; doi:10.3389/fpsyt.2025.1574448)
Supplement: Appendix 2 — Semi-Structured Questions: Questions used gather qualitative data during participants semi-structured interviews. [file DataSheet2.pdf]

HALTER Caregiver Interview Questions

1. Are there other caregivers for your child?
2. Do you attend a support group for parents of children with ASD?
3. What do you do for relaxation?
4. What are some stressors in your life?
5. What are some of your hobbies?
6. Do you spend time with other parents of children with ASD outside of HALTER?
7. How many hours a week do you think you spend on therapies specifically for your child's ASD?
8. Do you feel that there is an adequate variety of resources in your area for your child?
9. What are your sleep patterns?
10. Tell me what you do when you need a break from your child.
